# Disparities in Musculoskeletal Oncology

**DOI:** 10.1007/s12178-024-09925-8

**Published:** 2024-09-24

**Authors:** Abigail Koons, Elyse Smith, Jeffrey C. Stephens, Natilyn H. McKnight, Jennifer Barr, Izuchukwu K. Ibe

**Affiliations:** Department of Orthopaedic Surgery and Rehabilitation, University of Mississippi Medical Center, 2500 N State St, Jackson, MS 39216 USA

**Keywords:** Health disparities, Sarcoma, Musculoskeletal oncology, Disparities, Socioeconomic status, Adverse health outcomes, Cancer

## Abstract

**Purpose of Review:**

Disparities within the healthcare system serve as barriers to care that lead to poor outcomes for patients. These healthcare disparities are present in all facets of medicine and extend to musculoskeletal oncology care. There are various tenets to health disparities with some factors being modifiable and non-modifiable. The factors play a direct role in a patient’s access to care, time of presentation, poor social determinants of health, outcomes and survival.

**Recent Findings:**

In musculoskeletal oncologic care, factors such as race, socioeconomic factors and insurance status are correlated to advanced disease upon presentation and poor survival for patients with a sarcoma diagnosis. These factors complicate the proper delivery of coordinated care that is required for optimizing patient outcomes.

**Summary:**

Healthcare disparities lead to suboptimal outcomes for patients who require musculoskeletal oncologic care in the short and long term. More research is required to identify ways to address the known modifiable and non-modifiable factors to improve patient outcome.

## Introduction

Health disparities are inequalities in the healthcare experiences of individuals that exist as a result of differences associated with race, ethnicity, disability, gender and sexual identity, geographic location, income, education, insurance, and more [1]. Health disparities have allowed for the identification of a link between medical outcomes and social, economic, and environmental factors. The associated social conditions in which individuals live, learn, work, and play directly affect their ability to receive care in a complex medical system. Key factors are the obstacles and barriers created that contribute to the impairment of an individual(s) access to care. Examples include increased distance to care, language barrier or under/un-insured status [2]. Health disparities directly affect the treatment of acute as well as chronic diseases. For Chronic diseases, medication cost, health literacy, perceived discrimination, and poor insurance coverage are all an issue. All of these can lead to a lack of treatment, delay in the provision of care and a decrease in anticipated positive outcomes. These disparities are seen to play an influential role in various aspects of disease including incidence, prevalence, morbidity, and mortality [1].

Health disparities are related to many factors such as level of education, socioeconomic status, and geographical location. Medical information is often much easier to obtain and understand for individuals with a higher level of education [3]. Patients with a lower level of education may have a difficult time understanding the health information provided to them. Lower levels of education are associated with an increased risk of obesity, substance abuse, and injury [3]. Socioeconomic status and geographical location are two other important health disparities that contribute significantly to a patient’s overall health. These two factors play an important role in a patient’s access to nutritious food or affordable health care [4]. Without proper access to these necessities, staying healthy can be increasingly difficult for certain populations. Lower income families that lack health insurance are seen to have worse outcomes and a higher burden of disease [1].

**Fig. 1:**
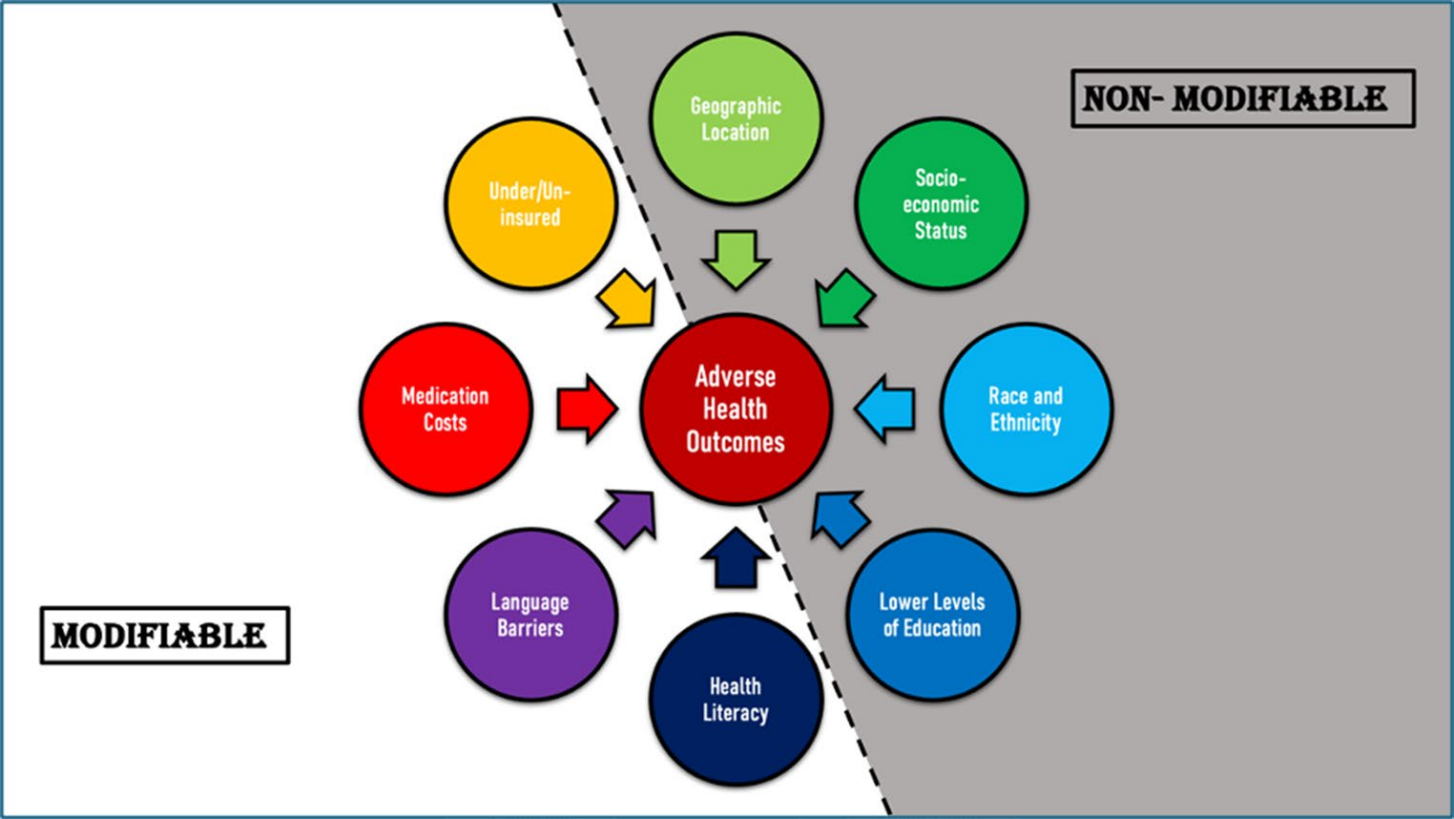
An outline of specific modifiable and nonmodifiable health disparities that exist and contribute towards adverse health outcomes including increasing incidence, prevalence, morbidity, and mortality along with a higher burden of disease seen in certain populations [1]

Race and ethnicity are important health disparities particularly because they are often associated with other social disadvantages that can lead to worse health. These disadvantages include poverty, residential segregation, limited education, lack of employment, and debt [2]. One not as commonly discussed health disparity includes the lack of diversity in clinical research and how this may lead to results not being applicable to all populations [5]. It is difficult to accurately assess potential adverse outcomes when initial clinical studies lack diversity. Research on health disparities and their direct effects on the health outcomes and well-being of patients could lead to a significant impact on the treatment of patients. Figure 1 identifies the most common health disparities and groups them into “modifiable” and “nonmodifiable” factors.

## Health Disparities in Orthopaedic Care

High-quality care is often inhibited by relevant healthcare disparities such as race, ethnicity, environment, socioeconomic status, and sex [2, 6]. Orthopaedics is no exception to this reality. Disparities within the field of orthopaedic surgery can be organized into factors related to patients, practitioners, and systems as a whole [7], and the effects of these disparities continue to be reported across healthcare.

### Patient-Related

Patient-related factors can be further divided into socio-demographic and clinical variables. One study retrospectively analyzed these variables in operative versus nonoperative management of calcaneus fractures. Socio-demographic variables including advanced age, female gender, Medicare insurance, lower socioeconomic status and minority race/ethnicity were found to have a statistically significant correlation to decreased utilization of open reduction and internal fixation. A similar statistically significant relationship was reported for clinical variables such as diabetes mellitus, peripheral vascular disease, drug and alcohol abuse, and psychosis. Another study observed that patients who are belonged to racial minorities, low educational and socioeconomic status, and public insurance policies demonstrated decreased use of outpatient orthopaedic care. Additionally, this study highlighted a significant association of the identified characteristics with increased use of emergency department care [7].

Sociodemographic variables also create orthopaedic healthcare disparities in the pediatric population. Factors such as race, household income, parental involvement, geographic location, and socioeconomic status consistently contribute to undesirable outcomes due to inadequate access to quality care [9]. Arant et al. reviewed a total of 36 sources detailing disparities in pediatric orthopaedic patient populations and identified several concrete results worth noting. Insurance status, race, and social deprivation were found to be associated with poor healthcare access which has been shown to contribute to delayed presentation. This then leads to increased time to diagnostic imaging and therefore delayed surgical remediation of time sensitive injuries [9].

### Practitioner-Related

Practitioner-related factors have demonstrated involvement in racial and social healthcare disparities. Unfortunately, minorities often experience implicit bias in healthcare. The term implicit bias describes a pervasive societal preference for specific groups. This bias is reflexively formed as a product of one’s perceptions and experiences, and in the case of a physician, it can have a negative effect on patient interaction by tainting communication, clinical assessment, and medical decision making [10].

Additionally, diagnostic reliability depends on setting of the practitioner assessing the injury. This alone lends itself to be a disparity. For example, two patients could present to the emergency department (ED) and a private outpatient orthopaedic clinic with identical knee injuries. The patient in the ED is given the broad diagnosis of joint derangement with the expectation that they would follow-up with the outpatient clinic for further diagnosis, while the clinic patient is diagnosed with an anterior cruciate ligament (ACL) tear and subsequently treated [7, 8]. The ED physician most likely gave the broad diagnosis with the expectation that the patient will follow-up with an orthopaedic clinic for diagnostic clarity. This becomes problematic when the patient does not make it to the follow up and the diagnosis is missed, delaying appropriate care. It has already been established that ED use is increased in those of greater disparity, so it can be expected that these same patients will be unable to attend private outpatient follow up [7, 8]. Policy change directed towards improvement of access to outpatient care would ideally improve overall care for patients who struggle with disparities as well as reduce healthcare expenditures.

**Fig. 2:**
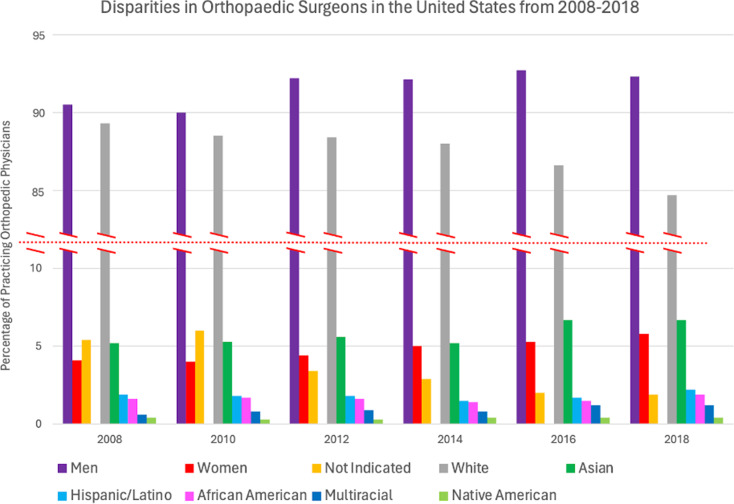
Self-identified sex and racial/ethnic breakdown of practicing orthopaedic surgeons in the United States from 2008 to 2018 [11]

Lack of diversity among orthopaedic surgeons has also been paralleled to healthcare disparities, demonstrated in Fig. 2 [11]. With these demographics in mind, this study made the following conclusions about gender and racial/ethnic disparities. Women’s access to and utilization of orthopaedic care are negatively impacted by gender-based practitioner disparities, as the majority are men. Women were also less likely to be referred after work-related shoulder injuries and showed lower rates of intervention specifically for arthroplasty and carpal tunnel release compared to males. It is suggested that these discrepancies can be attributed to the patient physician interaction. Several studies have been published regarding the relationship between patient-physician racial/ethnic concordance and patients’ decision to seek care for new health problems, chronic disease maintenance, and preventative medicine. Ma et al. emphasizes a significant positive correlation between racial/ethnic concordance and health care utilization. In other words, patients are more willing to visit physicians of the same race/ethnicity due to various factors such as improved communication secondary to language proficiency, avoidance of discrimination, more accurate risk assessment, and overall trust of providers with shared identity. Patients consistently report greater adherence to care plans and improvement in both outcomes and satisfaction when treated by physicians that are share the same race/ethnicity [12].

Other characteristics found to be related to underutilization included advanced age, any race other than White, low household income, and Medicare/Medicaid insurance policies [11]. The study also stated that African Americans experience more complications and poorer outcome scores than White patients undergoing the same orthopaedic procedures. According to Wright et al. sex and race are only two of many sociodemographic factors outside of patient control that influence orthopaedic outcomes.

### System-Related

System-related factors may also play a role in disparities experienced by orthopaedic patients. A study that examined the use of outpatient services in orthopaedic care demonstrated influence from both socioeconomic and demographic disparities. Specifically, government issued insurance as well as lack of insurance was associated with decreased use of outpatient clinical musculoskeletal care when compared to patients with private insurance practitioners [7]. This reinforces findings across many other studies that local surgeons are less likely to treat those presenting with public insurance or no insurance and instead tend to focus on privately insured patients. As a result, public insurance holders and the uninsured may be forced travel outside of their immediate areas in order to receive outpatient orthopaedic care [7]. This study also added to existing evidence that patients with public insurance utilize emergency services at higher rates than those with private insurance. This finding was true for all severities of musculoskeletal care [7]. Additionally, a study that compared ACL reconstruction incidence proportions derived that a private insurance company was more likely to be listed as the primary payer than a government insurance such as Medicare of Medicaid [6]. The conclusions of these two studies are consistent and highlight an obvious disparity in Orthopaedics; type of insurance plays a large role in access to care.

While various disparities have been discussed within orthopaedic care, it is crucial to acknowledge that no singular domain is independent of another. Disparities related to race, ethnicity, gender, socioeconomic status, insurance and many other social determinants of health are linked and oftentimes coexist in one patient [10]. While studies tend to assess such disparities individually, their effects can compound in actual patient’s circumstances as they are not mutually exclusive.

## Health Disparities in Oncologic Care

Cancer itself does not discriminate when it comes to prevalence across various populations. Secondary to the existing differences within populations, the burden of the disease is not uniform. These disparities facilitate the variations in metrics such as incidence, prevalence, morbidity, mortality, screening rates, and stage at diagnosis seen in statistical data regarding cancer in the United States [5, 13]. Individual populations that may experience such disparities can be grouped by race & ethnicity, gender identity, geographic location, socioeconomic status, and education level. While the overall mortality of cancer has reduced in the United States, this progress is not equally distributed, and trends do not remain consistent when broken down into these specific populations [13].

Cancer health disparities is a term becoming increasingly common in literature and can be defined as the measurable differences in cancer outcomes in various population groups [5, 13]. They create barriers to oncologic care which impacts the diagnosis, treatment, and proper delivery of care. The effects of cancer health disparities have been studied worldwide, and Rates of cancers including breast, colorectal, and prostate have been proven to vary greatly between high-income and low-income countries, geographic areas, and race/ethnic groups [14].

### Race and Ethnicity

**Fig. 3:**
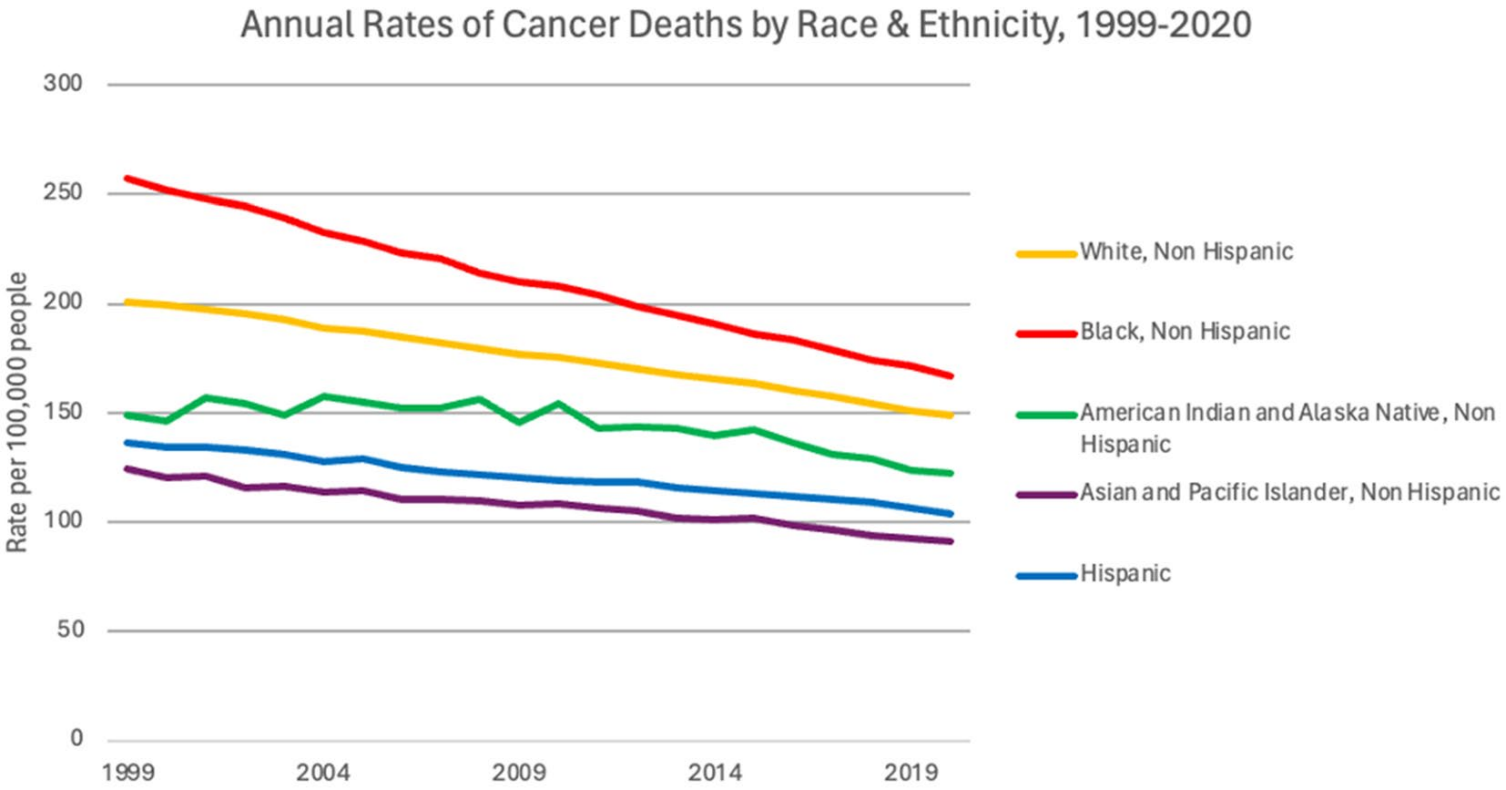
Annual rates of cancer deaths by race and ethnicity per 100,000 people shows that though rates have decreased over the past 20 years, differences still exist across races. Black individuals continue to be the race with the highest rates of cancer deaths [17]

Cancer health disparities related to race and ethnicity tend to lead the discussion in literature, with endless accounts of minorities experiencing disproportionate cancer burden. African American/Black individuals, for example, have a higher mortality rate than any other race for most cancers, as depicted in Fig. 3. As of 2022 data from the American Cancer Society, Black men had a 6% higher incidence but 19% higher mortality than White men. Black women actually had an 8% lower incidence; however, mortality was still 12% higher than White women [5, 15]. Following closely behind for mortality are Asian American, Latin American, and American Indian populations [16]. The reasoning for such discrepancies is multifactorial, however, research suggests that factors such as decreased access to healthcare, limited screening, socioeconomic status, and exposure to risk factors within these groups play a role [17, 18].

### Gender Identity

Men are at a 2x higher risk of dying from non-reproductive cancer than women. The reasoning is not yet understood; however, it is thought to be a combination of genetic influence as well as sex dependent response to therapy [14]. Behaviors related to gender identity can further this discussion. For example, the rate of tobacco and alcohol use has been found to be higher in LGBTQ youths than heterosexual youths, which is associated with increased risk of cancer [5]. This population has been found to be less likely to adhere to screening and have increased incidence of HIV and HPV associated cancers. Barriers for this specific population are furthered by discrimination and lack of sensitivity of health care professionals in both preventative care and treatment [19].

### Geographic Location

Geographic location can hinder appropriate oncologic care by means of poor healthcare access for screening [20] and exposure to conditions that increase risk of cancer [5]. Primary care practitioners employ screening tests for common treatable cancers, most notably colorectal, breast, cervical, and prostate. However, patients living in rural areas experience difficulty accessing such primary care services and therefore are not appropriately screened, leading to later stage at diagnosis [20–22]. Patients may live in communities lacking clean air or water which can lead to exposure to carcinogenic substances [5]. Poor work or housing conditions have specifically been found to cause exposure to asbestos and radon, both known carcinogens [20].

The concept of built environment has been identified to have influence on health outcomes, as the community in which one lives in can influence behaviors that increase risk of cancer. For example, lower income areas tend to lack affordable, nutritious food options and as well as safe areas for individuals to exercise. Subsequently, these populations boast high rates of obesity, which is a known risk factor for many cancers, with a 17% increased risk of cancer-specific mortality [23].

**Fig. 4:**
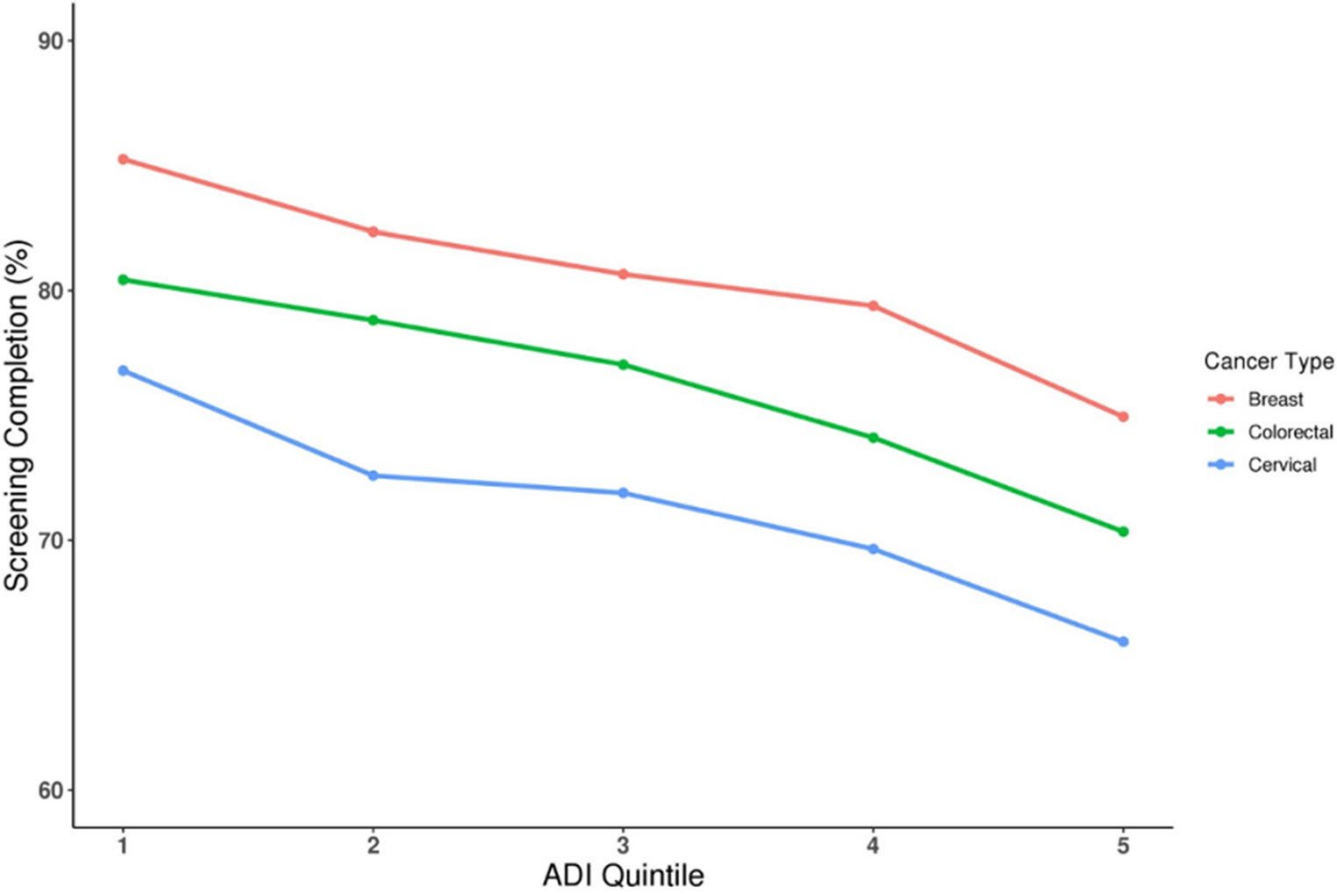
Area Deprivation Index (ADI) is a metric to describe the overall disparity within a zip code. High ADI indicates greater disparity and can be categorized 1–5. As ADI increases, screening completion rates of the area’s residents fall across all cancer types with routine screening [20]

Area deprivation index (ADI) is a measure of overall deprivation in an area based on 17 social determinants of health, assigned by zip code. Higher ADI indicates greater deprivation. A study correlating ADI to screening rates for breast, cervical, and colorectal cancer showed an inverse association with higher ADI having decreased odds of completing screenings, shown in Fig. 4 [21].

### Socioeconomic Status

**Fig. 5:**
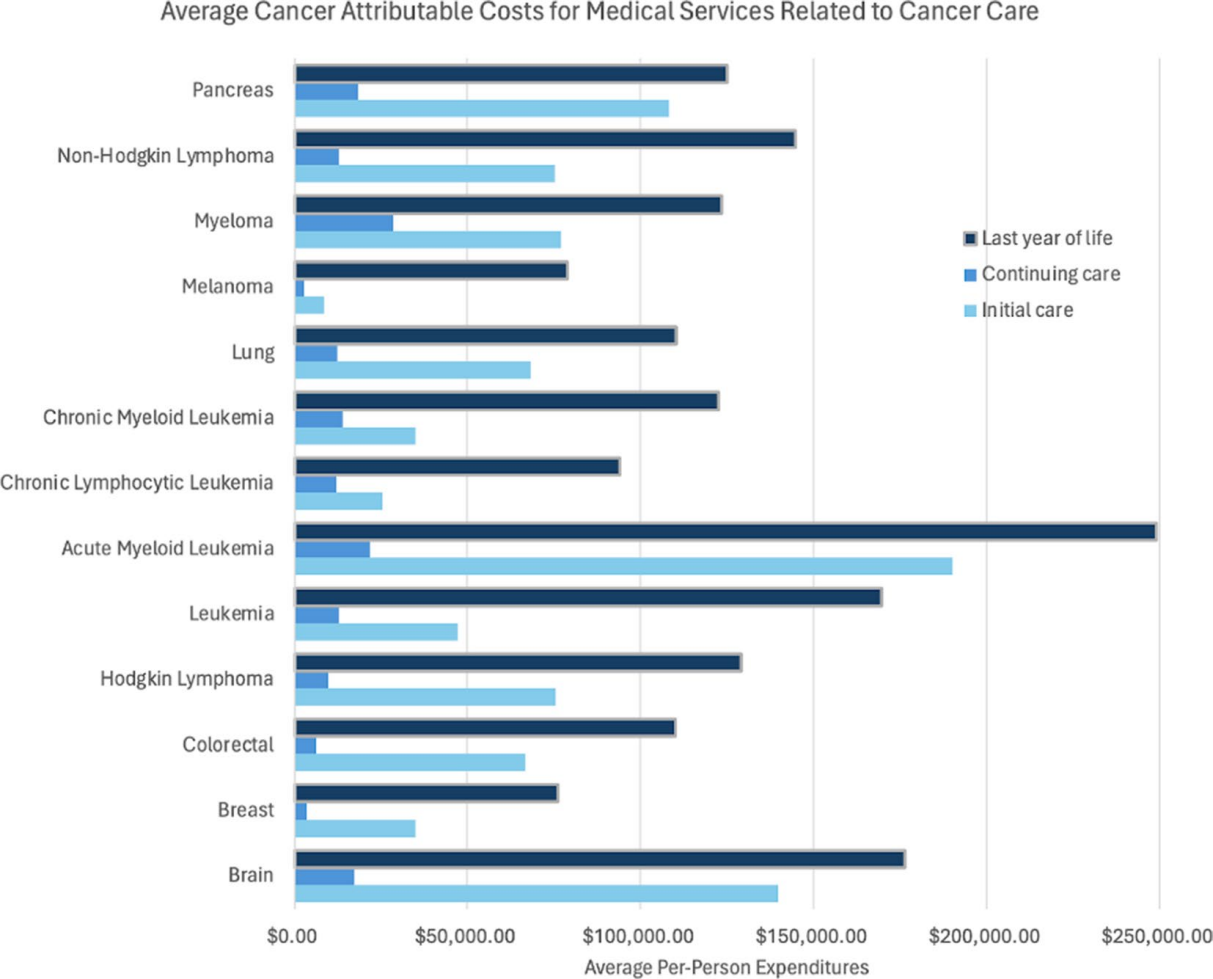
Cancer care is a tremendous financial burden. Acute Myeloid Leukemia is the most costly disease to treat, though all cancers depicted in this figure are costly and could be problematic for patients of lower socioeconomic status with poor financial stability and poor access to insurance [23]

Those of low socioeconomic status struggle to attain adequate oncologic health care for many reasons. This burden begins with inadequate funds to afford preventative screening tests, leading to later diagnosis. Those who are uninsured or Medicaid-insured have been proven to receive advanced stage diagnosis of cancer compared to those who are privately insured as well as decreased quality and quantity of treatment options [20]. Furthermore, cancer is known to be one of the most expensive medical conditions. Figure 5 illustrates the average costs at initiation of treatment, continuation of care, and within the last year of life for some of the most common cancer types. There is not a detailed breakdown of the cost of bone and joint cancer as it is not one of the most common cancer types, however literature notes that it can easily be upward of $100,000 [24].

The term “financial toxicity” has been coined to describe the negative impacts that the high out-of-pocket costs of cancer treatment can have on a patient’s life. Cancer is one of the most costly conditions in the United States due to expenses associated with treatments and hospitalizations. Various factors such as cancer type, financial status, and health insurance coverage impact the level of financial toxicity experienced by an individual patient. Additionally, cancer patients can experience difficulty maintaining the work schedule that they had prior to diagnosis as a result of both physical limitations and time limitations. This increased financial burden can leave patients with temptation to skip medication and treatment in order to maintain enough funds to afford their personal budget. Conversely, paying for the treatments can lead to lower quality of life, anxiety, depression, and disruption of personal finances in order to afford treatment [25].

### Education Level

Relationship between higher educational attainment and more favorable cancer related outcomes is evident in literature. There is a correlation between lower education level and lower health literacy. This can affect patients’ desire to seek appropriate cancer screening due to being unaware of the necessity, regardless of the patient’s ability to access or afford the testing. Lower educational achievement has also been linked to lower rates of appropriate therapies following diagnosis [20]. A study performed specifically on colorectal cancer patients showed that those with higher education are less likely to die before the age of 65 than those with less education, regardless of race or ethnicity [5].

### Lack of Diversity in Clinical Research

Cancer disparities are impacted by lack of diversity in participants of clinical research. Publishing work based on diverse participant pools would be most effective in establishing inclusivity and personalization of advancements in oncologic care applicable to all populations [5]. Unfortunately, research participant recruitment for cancer trials has not historically placed value in establishing a diverse group representative of the general population. Variations in demographics and clinical factors such as race, gender identity, socioeconomic status, age, and stage at diagnosis could lead to differences in effectiveness, tolerance and outcomes [13].

## Health Disparities in Musculoskeletal Oncology

In general, musculoskeletal oncology includes four broad groups of patients: those with metastatic disease to the bone, hematological malignancies with musculoskeletal manifestations, primary bone malignancies, and soft tissue sarcomas [26]. Of these, metastatic bone disease (MBD) is the most commonly seen contributor to the overall diagnostic number of musculoskeletal malignancies doled out annually [27]. The forerunner for hematological malignancies is multiple myeloma, a disease characterized by over proliferation of plasma cells within bone marrow [28]. The most commonly encountered primary bone malignancies in descending order of occurrence rate are osteosarcoma, Ewing sarcoma, and chondrosarcoma respectively. Lastly, soft tissue sarcomas impact the musculoskeletal system significantly, and those of the head and neck in particular are extensively represented in social determinants of health research [26]. Regardless of the diagnosis, overall patient survival and treatment type are two areas throughout literature that appear particularly susceptible to the negative effects of health disparities seen amongst the musculoskeletal oncology community.

### Effects of Health Disparities on Overall Survival

Primary bone malignancies (PBM) and soft tissue sarcomas (STS) are two pathologies that require early diagnosis and referral to a specialist if the patient has any chance of long-term survival. Treatment is often undertaken amongst a collaborative, multifaceted healthcare team typically only accessible at large tertiary care centers resulting in extensive barriers to treatment experienced by many patients of lower SES [24]. The need for travel, referrals to orthopaedic specialists, and long appointment wait times all impart further strain on an already vulnerable population. A study conducted by Braswell et al. found that early treatment with a multidisciplinary team mitigated the survival discrepancies seen amongst Black vs. White patients with non-metastatic high grade soft tissue sarcomas [29] highlighting the gravity of the impact that adequate and expedient care has on the outcomes of these patients.

One area directly implicated in the erection of these barriers to care is insurance type and its role in allowing or prohibiting access to quality and timely healthcare [30]. Longer survival time has been seen in patients with non-Medicaid/private insurance vs. that seen in uninsured patients or those with Medicaid [24]. One study even found that in a cohort of patients with PBM, lack of insurance and Medicaid were associated with an increased risk of metastatic disease being present at the time of initial diagnosis [31]. Advanced disease at time of presentation could explain the bleaker survival rates seen in these patient populations as they comprise a group long-since associated with lower SES. This draws on previously established findings linking health disparities with reduced rates of surgical intervention and adverse postoperative outcomes seen across orthopaedic care as a whole [6]. Recently the Journal of American Medical Association undertook a large-scale study using data from the SEER Census Tract-level SES Database to investigate the effects a patient’s SES, insurance status, and race/ethnicity have on the presence of metastatic disease at diagnosis in multiple histologically distinct sarcoma subtypes across a broad age range. Table 1 details the odds ratios of metastases at diagnosis calculated for each of the variables of interest in adults aged 20–65 years [32]. Interestingly, they found no associated risk between lower SES and the presence of metastases at diagnosis for the majority of the 25 STS subtypes evaluated. However, there was a statistically significant association between having Medicaid insurance or no insurance at all and the presence of metastases at diagnosis in these same populations underscoring the deleteriousness imparted by diagnostic delay on advanced stage at presentation in these patients. Additionally, having Medicaid or no insurance was found to be associated with an increased odds of metastases at diagnosis in 6 of the 8 subtypes evaluated. Osteosarcoma and Ewing sarcoma were the only 2 subtypes not associated with insurance status. The lack of associated risk between lower SES and insurance status on the presence of metastases at diagnosis for the osteosarcoma and Ewing sarcoma subtypes implies factors other than diagnostic delay and barriers to timely treatment may be responsible for the more advanced presentation of these disease types [32].

**Table 1:**
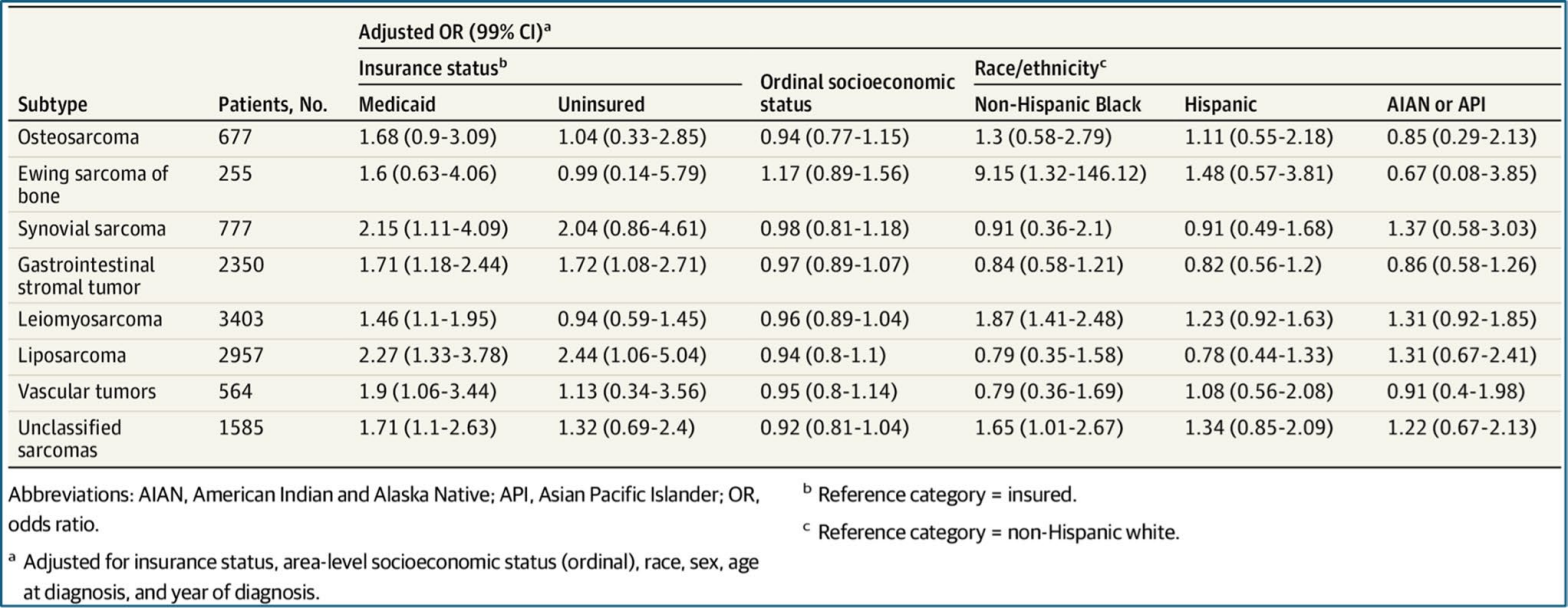
Multivariable adjusted odds ratios for metastasis at diagnosis in adults aged 20-65y at diagnosis, by insurance status, stratified by sarcoma subtype: Surveillance, Epidemiology, and end results 16 registries, 2007–2015

Referrals to orthopaedic specialists also play a direct role in patient survival, with longer wait times resulting in further progression of disease. This delay in treatment was found to be exacerbated by patients with Medicaid insurance when compared to their privately insured cohort [24]. Additionally, MBD was found to have a higher incidence rate amongst those with lower SES contributing to the idea that inadequacies in access to reliable screening and timely treatment interventions play a role in the discrepancies of survival rates seen amongst patients suffering from a multitude of other primary cancer types [20, 27]. While these inadequacies definitely contribute to the increase in incidence of MBD and metastases at diagnosis seen amongst patients of lower SES, disparities affecting geographic location and a patient’s-built environment could also be at play and should be considered when treating patients from socioeconomic, racial, and ethnic groups at greater risk of experiencing these barriers to adequate healthcare.

### Effects of Health Disparities on Treatment Type

Historically, limb amputation was considered the standard of care for patients diagnosed with PBM. These surgeries resulted in severe physical deformity with little to no evidence of mortality benefit ultimately leading to the advent of newer treatment methods including limb salvage and reconstruction [30]. The emergence of these newer, less disfiguring surgical techniques has resulted in better functional outcomes and overall quality of life for patients being treated for diseases under the musculoskeletal oncology umbrella. Despite these surgical advancements reducing the widespread use of amputation as a mainstay of treatment, studies are still consistently finding higher rates of amputation amongst minority populations [30]. As discussed previously, insurance status has long since been an indicator of someone’s SES thus having a direct effect on the outcomes of patients suffering from musculoskeletal malignancies. Smartt et al. found that patients with Medicaid insurance were twice as likely to undergo amputation surgery than those with non-Medicaid insurance in the treatment of both PBM and STS [24]. Whether this difference is due to advanced disease stage at presentation or limited access to care at facilities capable of performing complex limb salvage therapy begs to be addressed. Regardless, the hypothesis that lower SES plays a role in the type of treatment received by patients with PBM and STS fits well into documented survival discrepancies between SES groups [30].

Racial and ethnic factors have also been implicated in treatment disparities seen amongst patient populations. Lapica et al. found a significant difference in amputation rates between Hispanics and non-Hispanics with Hispanics more likely to receive amputation vs. limb salvage therapy [30]. Surprisingly, this same study also found no association between amputation rates and Black and White race in the treatment of osteosarcoma, seeming to contradict current literature in which Black race was found to be an independent predictor of amputation in the treatment of various limb-compromising diseases including STS [30]. In regard to operative vs. non-operative interventions, one study found that Black and Asian/Pacific Islander patients were less likely than White patients to undergo surgical treatment for PBM. Additionally, non-Hispanic Blacks diagnosed with multiple myeloma have been found to have a lower utilization rate of novel therapeutic drugs and autologous stem cell transplantation (ASCT) than non-Hispanic Whites [28]. With overall survival rates doubling since the emergence of these new treatment modalities, the reduced use of these therapies amongst a specific racial/ethnic group suffering from multiple myeloma is another grave illustration of disparities in care seen amongst minority groups [28]. These findings reinforce the idea that racial and ethnic factors along with their associated SES disparities are also at play in the treatment of these malignancies [31].

## Conclusion

Broadly speaking, a person’s unique social determinants of health (SDoH) represent the complex interplay between the social and physical environment into which that person is born and lives. This interplay directly influences the ability to access resources that increase a person’s overall quality of life [20]. SDoH are the key factors that imbue groups of people with the healthcare disparities seen ubiquitously across all medical specialties. As sarcomas are notorious for displaying few early recognizable signs and symptoms, musculoskeletal oncology patients represent a population particularly vulnerable to delays in diagnosis and prolonged time to treatment [32]. Barriers to adequate and timely care are erected through modifiable and non-modifiable disparities all implicated in adverse health outcomes of those affected. Studies of state healthcare spending have shown that those states more financially inclined towards improvement in social services (rather than government-funded medical programs like Medicaid and Medicare) displayed much better health outcomes amongst their populations than those that were not [20]. This finding illustrates the need for a comprehensive approach to mitigating healthcare disparities; one that focuses on formulating treatment plans with shared decision-making between the patient and physician; one that evaluates the patient within their unique living environment taking into account all facets of the patient that could affect their diagnosis and treatment. Notorious for displaying few early recognizable signs and symptoms, sarcomas and musculoskeletal malignancies alike put their patient populations at a considerably heightened risk for delayed diagnosis and time to treatment [32]. In order to mitigate the gap in outcomes seen amongst those from lower SES, healthcare professionals must identify and quell any unconscious resistance that could directly impede a patient’s access to adequate care. They must be willing to address both system-related and personal behaviors that may be contributing to these disparities including addressing institutional discriminations, examining personal biases, and participating in activities that work towards more diverse and equitable clinical practices and research endeavors [13].

## Data Availability

No datasets were generated or analysed during the current study.
